# Altered DMN functional connectivity and regional homogeneity in partial epilepsy patients: a seventy cases study

**DOI:** 10.18632/oncotarget.20575

**Published:** 2017-08-28

**Authors:** Chong-Yu Hu, Xiaoping Gao, Lili Long, Xiaoyan Long, Chaorong Liu, Yayu Chen, Yuanyuan Xie, Chujuan Liu, Bo Xiao, Zhe-Yu Hu

**Affiliations:** ^1^ Department of Neurology, Hunan Provincial People's Hospital, Changsha, Hunan, China; ^2^ Department of Neurology, Xiangya Hospital of Central South University, Changsha, Hunan, China; ^3^ Department of Rehabilitation, Hunan Provincial People's Hospital, Changsha, Hunan, China; ^4^ Department of Clinical Research and Teaching, The First Hospital of Changsha, Changsha, Hunan, China

**Keywords:** partial epilepsy, regional homogeneity, default mode network, resting state, BOLD-fMRI

## Abstract

**Purpose:**

Clinically diagnosed partial epilepsy is hard to be functionally diagnosed by regular electroencephalograph (EEG) and conventional magnetic resonance imaging (MRI). By collecting transient brain regional signals, blood oxygenation level-dependent (BOLD) function MRI (BOLD-fMRI) can provide brain function change information with high accuracy. By using resting state BOLD-fMRI technique, we aim to investigate the changes of brain function in partial epilepsy patients.

**Methods:**

BOLD-fMRI scanning was performed in 70 partial epilepsy and 70 healthy people. BOLD-fMRI data was analyzed by using the Regional Homogeneity (ReHo) method and functional connectivity of Default Mode Network (DMN) methods. The abnormal brain functional connectivity in partial epilepsy patients was detected by Statistical Parametric Mapping 8 (SPM8) analysis.

**Results:**

Compared to healthy group, epilepsy patients showed significant decreased ReHo in left inferior parietal lobule/pre- and post-central gyrus, right thalamus/paracentral lobule/Cerebellum anterior and posterior Lobe, bilateral insula. The DMN functional connectivity regions decreased significantly in right uncus, left Inferior parietal lobule, left supramarginal gyrus, left uncus, left parahippocampa gyrus, and left superior temporal gyrus, in epilepsy patients, compared to healthy controls.

**Significance:**

This study clarified that both ReHo and functional connectivity of DMN decreased in partial epilepsy patients compared to healthy controls. While left inferior parietal lobule was detected in both ReHo and DMN, many other identified regions were different by using these two BOLD-fMRI techniques. We propose that both ReHo and DMN patterns in BOLD-fMRI may suggest networks responsible for partial epilepsy genesis or progression.

## INTRODUCTION

Epilepsy is a global problem, and more than 60 million people are affected globally in 2011 [1–2]. It is one of the most common chronic serious neurologic diseases with a incidence rate of 0.4%–1% globally and 0.6%-1% in developing countries [3]. There are two versions of epilepsy categorization: the International League Against Epilepsy (ILEA) 1981 criteria and ILEA 2010 criteria. Currently, version 1981 is commonly used in clinical settings [4]. It is the foundation of 2010 classification and 2017 novel classification [5]. According to 1981 classification, partial epilepsy refers to focal or localization-related seizures with temporal focal spike and wave complex on the electroencephalogram (EEG), which differs from the generalized types of epilepsy [6]. Cognitive dysfunctions, including dysmnesia, language barrier and emotional disturbance, are common clinical manifestations [7–8]. In some of the partial epilepsy patients, conventional MRI can locate the abnormal brain lesion. However, in other part of the patients, the causes of epilepsy are complex and unclear. In this study, we investigated this group of patients whose conventional MRI have no abnormity.

While EEG excels in detecting the brain electric activities signals longitudinally, fMRI focus more on the spatial and functional changes. Based on the change of local oxygen consumption and blood flow in neuronal activation, BOLD-fMRI can detect the transient local signals. In resting state, specific brain region could be stimulated and generate more oxyhemoglobin which result in relatively lower deoxyhemoglobin level and increase T2* weighted phase signal [9]. Currently, simultaneous EEG and fMRI are used to localize the abnormal areas in patients’ brain, providing useful information for pre-surgery assessment [10]. Task-related BOLD-fMRI is much more frequently used to locate the lesions in language, memory and sensorimotor regions [11–12], which are important in pre-surgery planning. Compared to task-related fMRI, the resting state fMRI based on resting state BOLD signal is easier and more consistent [13].

Raichle, et al. [14], define the baseline state of brain activity as the resting state (awake and lying quietly with eye closed). In resting state, the ratio of oxygen used by brain to oxygen delivered by flowing blood (OEF) is uniform in some brain regions. This network distributes separately but highly functional related, including precuneus (PCu)/posterior cingulated cortex (PCC), medial prefrontal cortex (MPFC), ventral anterior cingulated cortex (cAVV), hippocampus, gyrus, parietal lobe, temporal lobe, etc. This network is called the Default Mode Network (DMN) and it is responsible for the readiness and alertness to changes in the external and internal environment [15]. During human behaviors and brain activities, local deviations in the OEF in DMN could be captured by BOLD-fMRI. For example, in temporal lobe epilepsy (TLE) patients, the BOLD signal increased in mesial temporal lobe, but decreased in DMN during seizure interval [16–17].

Regional homogeneity (ReHo) is another vital technique in resting state fMRI analysis [18]. Unlike functional connectivity, ReHo analyzes the synchronization during time series in neuronal activity regions. Abnormal ReHo signal indicates a neuron connection disorder [19]. Using ReHo alone, Yang, et al., suggested positive correlations between ReHo values in precuneus/PCC and supplementary motor area (SMA). Combined with DMN, Zeng, et al, identified a decreased ReHo pattern in DMN, which might be responsible for impairment of cognitive function in mesial temporal lobe epilepsy and hippocampus sclerosis (mTLE-HS) patients [20–21].

In this study, we collected 70 patients and performed both DMN and ReHo analysis. Compared to healthy group, left inferior parietal lobule was detected in both ReHo and DMN. But many other regions identified by ReHo and DMN were different. So, we proposed that by using these two BOLD-fMRI techniques, both ReHo and DMN may suggest network responsible for partial epilepsy genesis or progression.

## MATERIALS AND METHODS

### Participants

#### Patients

BOLD-fMRI data were gathered in 70 right-handed partial epilespy cases in Xiangya Hospital and Hunan Provincial People's Hospital between January 2010 and June 2014. Of all patients, 33 are females and 37 are males. The diagnosis of partial epilepsy was based on clinical symptom, EEG and MRI test. The inclusion criterion:

- According to 1981 International League Against Epilepsy (ILEA) classification, partial epilepsy has seizure ≥ twice per year;
- Age of 14 to 50 years old (in clinical setting, brain development in people with 14-year old has reached adult level, so epilepsy patients at 14 visit department of adult neurology);
- Educational levels higher than primary school graduation;
- No abnormality in routine conventional MRI
- Exclude patients with other transient brain dysfunction, such as syncope, transient ischemia attack (TIA), hysteria, and migraine;
- Exclude patients with serious physical or mental diseases, patients with alcohol or drug abuse, patients with long-term usage of drugs except anti-epileptic drugs, or patients with family history of mental illness.

#### Health controls

seventy right-handed healthy people whose age, gender and education levels were matched with recruited patients were served as control (Table 1). This group of healthy people has no systematic neurology disease or symptoms and no family history of neurology disease. Routine conventional MRI findings are normal.

**Table 1:** Demographics for patients (n = 62) and healthy controls (n = 64)

		Participants		
variables	Levels	Patients (*n* = 62)	Controls (*n* = 70)	*p*-value
Age		27.9 ± 8.3	29.1 ± 7.5	0.39**
Sex	Male	32 (51.6%)	35 (54.7%)	0.73†
	Female	30 (48.4%)	29 (45.3%)	
Education level	Low education level	5 (8.06%)	7 (10.94%)	0.80‡
	High School or *TSS	30 (48.4%)	28 (43.2%)	
	College or above	27 (43.6%)	29 (45.3%)	

Note: *TSS is technique secondary school. **t-test was performed to calculate *p*-value for age at diagnosis; †for variable sex, chi-square test was performed; ‡ for variable educational level, low education level includes no education, primary school education and junior school education. Chi-square test was performaed to evaluate the association of education level with partial epilepsy.

This study was approved by the institutional review Board of Health and Family Planning Commission of Hunan Provincial People's Hospital. The informed consent was obtained from each participant.

### MRI data acquisition

Each participant lay supine with eye closed and body still. Head was fixed by a matched hood. During scanning, participants should awake and be relaxed without thinking. Several strategies were used: 1) To ensure patient to be awake during scanning, every recruited patient was asked if he or she fell asleep. If his or her answer is YES, repeated scanning was performed. 2) To make fMRI output comparable, every subject was instructed to follow the same steps: supine, stay static, close eyes, not thinking, and not sleep. 3) The scanning time was about 7 minutes per patient, and during this period of time all patient and healthy controls should keep awake.

Images were acquired using 3.0T scanner (Siemens magnet on trio, A Tim System, German) in Hunan Provincial People's Hospital. We performed location image scanning first, and then T1 scanning with following parameters: TR = 1900, TE = 3.4 ms, fov = 24 × 24 cm, matrix = 256 × 256, thickness/gap = 1/0.5 mm, 160 volumes. In this study, we did not detect any abnormality in our participants. Then, we continued to use T1 as located image to perform resting-state fMRI. The echo-planar imaging sequence has the following parameters: TR = 3000, TE = 30 ms, fov = 24 ×24 cm, matrix = 64× 64, flip angel = 90°, thickness/gap = 3/1 mm, 36 volumes, scanning time = 7 min 08 sec.

### Data pre-processing

MatLab (The Math Works Inc. Natick, Massachusetts, USA), MRIconvert (http://lcni.uoregon.edu/jolinda/MRIConvert/), SPM8 (Statistical Parametric Mapping, http://www.fil.ion.ucl.ac.uk/spm/), and RESTing state fMRI data analysis toolkit (REST, by Song Xiaowei, http://resting-fmri.sourceforge.net) were used to pre-process MRI data and perform statistic analysis. The detailed steps were described as follow:

1. Converting acquired MRI figures to .img format by using MRIconvert software. Because magnetic field need time to become steady and patient also need some time to accommodate environment, so the first five time points MRI figures were deleted. The next 135 time-points were analyzed.
2. To eliminate head motion-induced noise, SPM8 was then used to evaluate the linear shifting of head on x, y, z axis, and the rotation of head around x, y, z axis. Only patients with linear shifting distances less than 1cm and rotation angles less than 1° were recruited.
3. Because anatomic structures of subjects’ brains were different from each other, so their brain fMRI figures need to be normalized spatially. All testing fMRI figures were normalized by using standard SPM8 EPI template. Figure data were re-sampled based on a volume of 3 × 3 × 3 mm^3^ as a unit.
4. To reduce spatial noise and increase signal-to-noise ratio (SNR), we used 8×8×8 mm^3^ FWHM (full-width at half maximum) to smooth fMRI figures and eliminate the bias caused by spatial normalization.
5. Resting State fMRI data analysis toolkit (REST) were applied to remove baseline shift and filter noisy waves (f < 0.01Hz or f > 0.08Hz). Low-frequent baseline shift (f < 0.01Hz) was generated by the heat noise brought by coil preamplifier. High-frequent physiologic noise (f > 0.08Hz) was generated by breathing and heart-beating, etc.

### Regional homogeneity analysis

MatLab and RESTing state fMRI data analysis toolkit (REST), and MRIconvert were used to perform Regional Homogeneity (ReHo) analysis.

In fMRI, brain active region was formed by multiple connecting voxels in space. ReHo assumes selected voxel to be transiently similar to adjacent voxel. These ReHo voxels exhibit the same changes at the same time sequence. Here, we calculated the Kendall's coefficient concordance (KCC) to evaluate the similarity between the selected voxel and the adjacent voxel [18]. In Matlab, REST package was used to calculate KCC values.

$$W=\frac{å\;{\left(R_{i}\right)}^{2}-n{\left(\bar{R}\right)}^{2}}{\frac{1}{12}K^{2}(n^{3}-n)},\text{ where }n=135$$

W measures the KKC value for selected voxel, ranging from 0 to 1. Ri is the sum of rank for time point i. $\bar{R}=(n+1)\times K/2$, indicating the average value of Ri. K is the number of calculation units (the number of adjacent voxels to form a minimal ReHo calculation unit). Here, K = 27, one selected voxel and 26 adjacent voxels.

, where *n* = 135

### Functional connectivity analysis

PCC and PCu are two adjacent areas, mainly located in Brodmann 7 (BA7) and 31 (BA31) regions. Here, we focused on BA31 region because Raichle, et al., reported that the DMN PCC/PCu localizes in BA31 region [14]. We chose a seed zone (circle) with a radius of 10 mm from the PCu center at coordinate point = (–12, −47, 32) [22]. MetLab software was used to calculate the correlation coefficient of the seed zone compared with the whole brain in patients and in healthy controls. We then used Fisher's r-to-z conversion to transfer the correlation coefficient (r) to z statistics:

$$Z=\frac{1+r}{1-r}$$

Then, based on normal assumption, z statistics were adopted in following statistic analysis. *T*-test was performed to compare the difference of correlation coefficient between patients and healthy controls. According to *t*-test statistic results, differences of DMN functional connectivity region between patients and healthy controls were detected By using RESTing state fMRI data analysis toolkit (REST).

### Statistic analysis

Numeric variables were summarized as mean (standard deviation). Categorical variables were reported as counts (percentage). To compare continuous variables (age and psychological assessment) that appear to have symmetric distributions between the two groups, analysis of variance was employed. To compare categorical variables between two treatment group, chi-square test or fisher's exact (*n* < 5) were performed.

One-sample *t*-test was performed to analyze the functional connectivity (*z* values) in each group. Two-sample *t*-test was performed to compare the ReHo (W values) and functional connectivity values for matched case and control groups. A cluster > 50 voxels with *p*-value < 0.001 was considered as significant. All statistical analyses were conducted using MatLab.

## RESULTS

### Demographics

Of the 70 patient cases included for analysis, 8 cases were removed because of ineligible MRI quality. Among the remaining 62 cases, 30 were females and 32 were males. Age (27.9 ± 8.3 yr) ranged from 14 yr to 48 yr. Of 70 controls, 6 cases were removed due to head motion or data quality reasons. Among the remaining 64 healthy controls, 29 were females and 35 were males. Age = 29.1 ± 7.5, ranged from 15 to 45. As demonstrated in Table 1, no significant age, gender or educational disparity could be detected between case and control group.

Compared to healthy controls, the numerical depth, language fluency test (accuracy number) and logic memory in patients were significantly lower (*p* < 0.001). SDS score and SAS score in epilepsy patients were much higher than control group (Supplementary Table 1).

### DNM functional connectivity comparison in epilepsy patients and healthy controls

**Figure 1:**
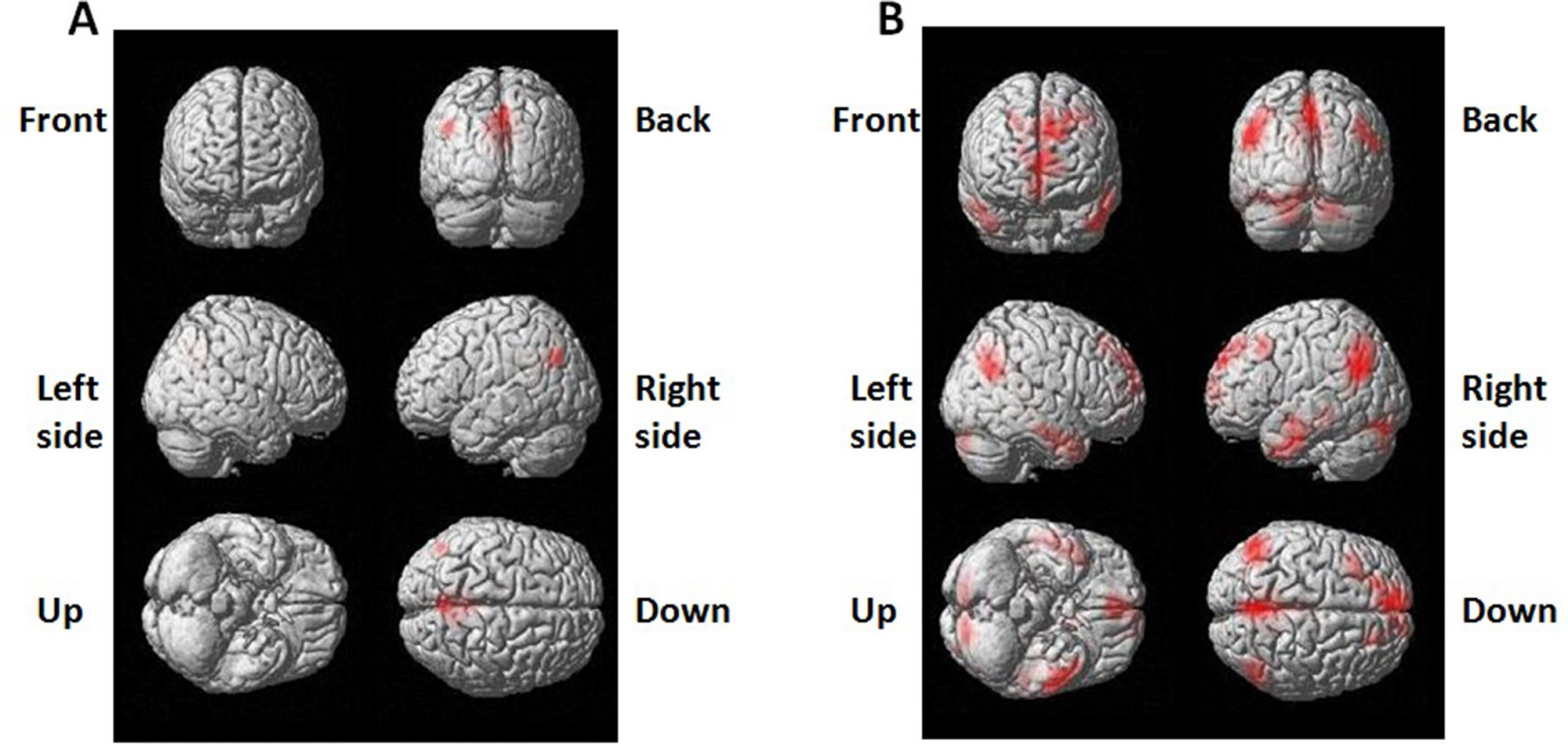
3-dimensional projection images for DMN functional connectivity regions in (**A**) epilepsy patients and (**B**) healthy control. Red color indicates DMN regions.

By using resting state BOLD-fMRI scan and the functional connectivity analysis, we analyzed DMN regions in partial epilepsy patients and healthy controls. In healthy controls, the DMN functional connectivity regions covered left Pcu /PCC and angular gyrus, cingulated gyrus in epilepsy patients (Figure 1A). In healthy controls, the DMN functional connectivity regions included Pcu/PCC, right angular gyrus, bilateral medial frontal lobes and temporal lobes in healthy controls (Figure 1B). These findings further verified the existence of DMN at resting status. The DMN functional connectivity region in partial epilepsy patients was much smaller than the size of DMN functional connectivity regions in healthy controls. Detailed information was demonstrated in Supplementary Tables 2 and 3.

**Figure 2:**
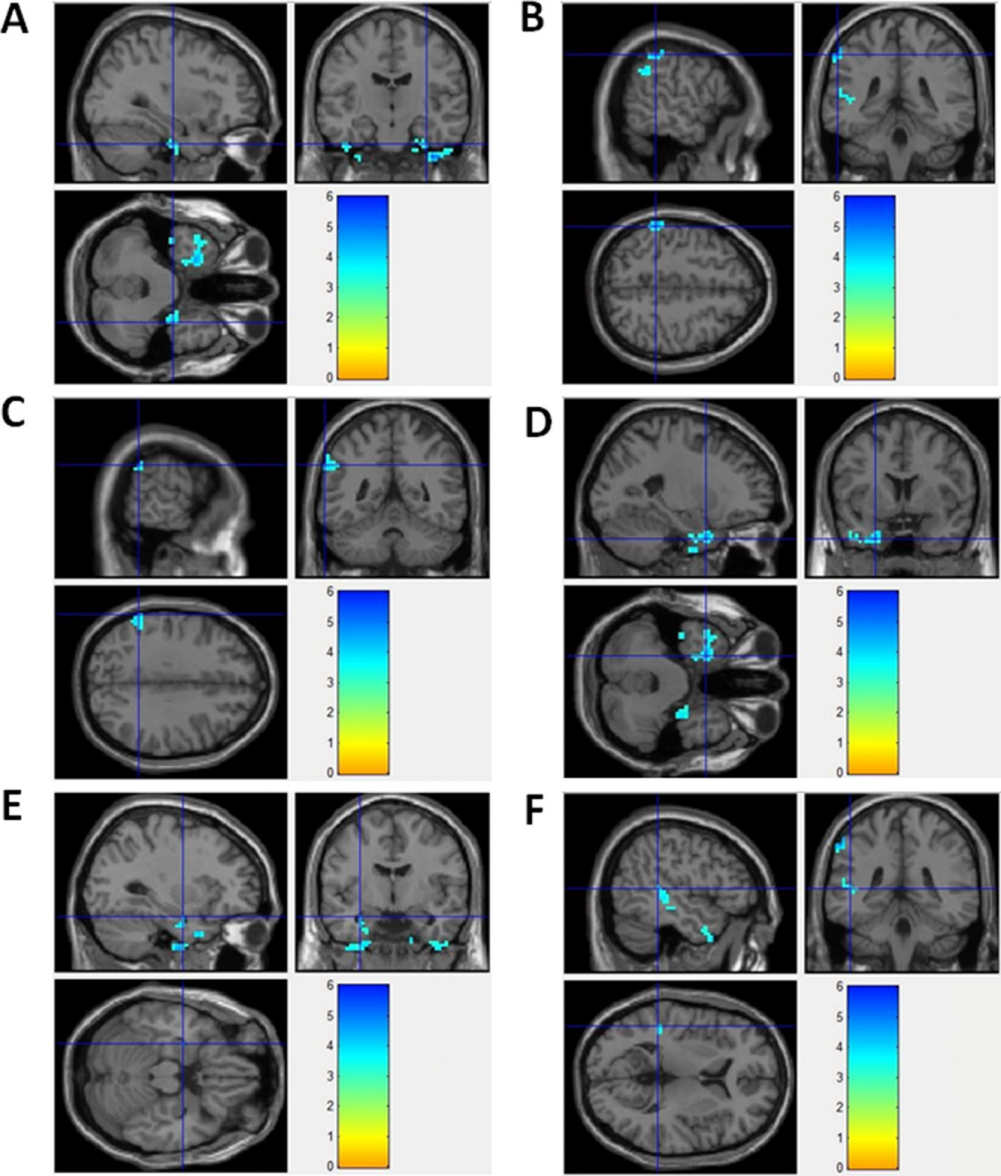
Brain regions with decreased DMN functional connectivity in epilepsy patients (**A**) Right Uncus; (**B**) Left Inferior Parietal Lobule; (**C**) Left SupramarginalGyrus; (**D**) Left Uncus; (**E**) Left ParahippocampaGyrus; (**F**) Left Superior Temporal Gyrus

In this study, seed zone was chosen (in DMN region) with a radius of 10 mm from the PCu center at coordinate point = (–12, −47, 32). MetLab software was used to calculate the correlation coefficient of the seed zone compared with the whole brain in patients and in healthy controls. *T*-test was performed to compare the difference of correlation coefficient between patients and healthy controls. Compared to healthy controls, the functional connectivity regions decreased significantly in epilepsy patients. Figure 2 showed the brain regions with decreased DMN functional connectivity in epilepsy patients, including right uncus, left Inferior parietal lobule, left supramarginal gyrus, left uncus, left parahippocampa gyrus, and left superior temporal gyrus. No increased DMN functional connectivity was observed in epilepsy patients.

Detailed information was demonstrated in Table 2. Supplementary Figure 1 showed the 3-dimension projection images for decreased DMN functional connectivity regions in epilepsy patients, compared with healthy controls. Supplementary Figure 3 demonstrated the decreased functional connectivity in projection map on axial brain image in epilepsy patients. In epilepsy patients, functional connectivity-decreased regions partly located in DMN regions, such as parietal lobe and temporal lobe. Other part of functional connectivity-decreased regions located outside of DMN regions.

**Table 2:** Brain regions with decreased DMN functional connectivity in epilepsy patients

					Coordinate			
Pixel size	L/R	Brain region	T statistics	Z statistics	x	y	z	BA region
135	R	Uncus	4.10	3.67	33	–15	–36	20
66	L	Inferior Parietal Lobule	4.95	4.35	–57	–39	48	-
	L	SupramarginaGyrus	4.37	3.87	–63	–48	33	40
107	L	Uncus	4.71	4.10	–24	6	–36	-
	L	ParahippocampaGyrus	4.18	3.72	–30	–6	–21	-
60	L	Superior Temporal Gyrus	3.89	3.45	–48	–39	6	-

In this study, all epilepsy patients had conscious disorders. For these patients, DMN was unable to maintain wakefulness and perform surveillance and self-examination for internal and external environment. So, we supposed that the decreased DMN functional connectivity could be the mechanism for conscious disorders in these patients.

### Comparison of ReHo in epilepsy patients and healthy controls

**Figure 3:**
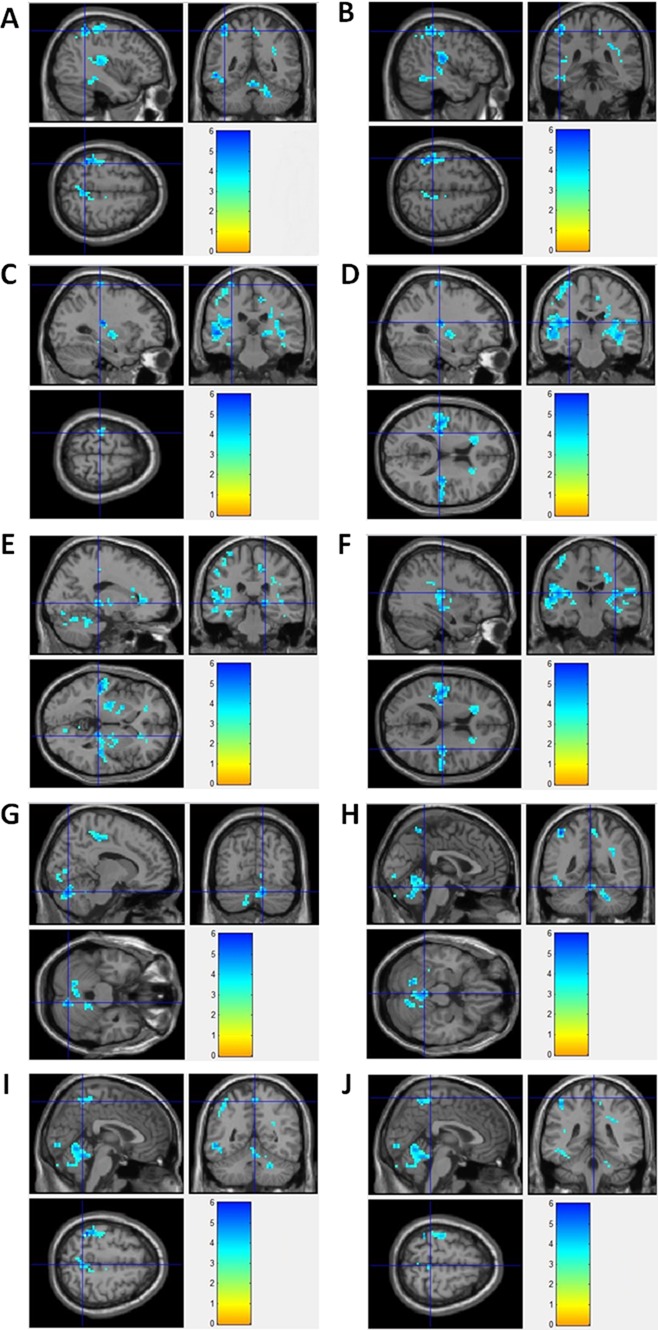
fMRI imagines for brain regions with decreased ReHo in epilepsy patients (**A**) Left Inferior Parietal Lobule; (**B**) Left PostcentralGyrus; (**C**) Left PrecentralGyrus; (**D**) Left Insula; (**E**) Right Thalamus; (**F**) Right Insula; (**G**) Right Cerebellum Posterior Lobe; (**H**) Right Cerebellum Anterior Lobe; (**I**) Right Precuneus; (**J**) Right Paracentral Lobule.

By using resting state BOLD-fMRI scan and ReHo analysis, no increased ReHo region was detected in this study. Instead, we found that the decreased ReHo regions covered left inferior parietal lobule/pre- and post-central gyrus, right thalamus/paracentral lobule/cerebellum anteriorand posterior Lobe, bilateral insula in epilepsy patients, compared to healthy controls (Figure 3). Detailed information for the decreased ReHo regions in epilepsy patients was described in Table 3. Supplementary Figure 2 showed the 3-dimension projection images for decreased ReHo regions in epilepsy patients, compared with healthy controls. Figure 3 showed the sagitta, coronal and axisal plane of the 3-D projection of ReHo map.

**Table 3:** Brain regions with decreased ReHo in partial epilepsy patients

					Coordinate			
Pixel size	L/R	Brain region	T statistics	Z statistics	x	y	z	BA region
189	L	inferior parietal lobule	7.36	5.23	–39	–48	57	-
	L	postcentralgyrus	6.34	4.71	–45	–36	54	40
	L	precentralgyrus	5.54	4.19	–30	–27	69	4
558	L	insula	6.41	5.07	–30	–24	15	13
302	R	thalamus	6.23	4.81	18	–30	0	-
	R	insula	6.09	4.51	36	–21	15	13
	R	insula	5.88	4.38	42	–24	-3	13
335	R	Cerebellum Posterior Lobe	6.45	4.72	12	–72	-27	-
	R	Cerebellum Anterior Lobe	6.30	4.62	0	–45	-21	-
	R	Cerebellum Anterior Lobe	5.79	4.36	3	–60	-9	-
57	R	Cerebellum Anterior Lobe	5.64	4.28	24	–45	-36	-
	R	Cerebellum Anterior Lobe	4.57	3.59	18	–42	-27	-
89	R	precuneus	5.39	4.12	3	–51	57	7
	R	paracentral lobule	4.42	3.54	3	–39	63	4

## DISCUSSION

At resting state, synchronous low frequency fluctuations (LFFs) exist in multiple related brain regions. So, when pathological changes occur in some of these regions, we can detect abnormal LFFs. Functional connectivity and ReHo are two primary techniques at resting state for pathological brain region analysis. In this study, we performed these two analyses and found that compared to healthy group, epilepsy patients showed significant decreased ReHo in left inferior parietal lobule/pre- and post-central gyrus, right thalamus/paracentral lobule/Cerebellum anterior and posterior Lobe, bilateral insula. The DMN functional connectivity regions decreased significantly in right uncus, left Inferior parietal lobule, left supramarginal gyrus, left uncus, left parahippocampa gyrus, and left superior temporal gyrus, in epilepsy patients, compared to healthy controls. No increased ReHo or DMN regions were identified in epilepsy patients.

In some studies, decreased ReHo was found to locate in regions of the DMN in epilepsy patients, such as children with new-onset drug-naïve benign epilepsy with centrotemporal spike (BECTS) and mTLE [20–21]. Here, we found that the decrease ReHo region, left inferior parietal lobule, localized in decreased DMN region. However, we still found many other identified regions to be different between ReHo and DMN patterns. Therefore, we proposed that ReHo and DMN patterns in BOLD-fMRI might suggest partial epilepsy genesis, progression or other implications. For example, DMN alteration in TLE may be related to GABAergic and glutamatergic dysfunction [23], suggesting a implications of DMN in TLE pathophysiology and treatment surveillance [16]. Moreover, researchers implied that the decreased DMN connectivity might be diverse among different seizure types [24]. Here, due to the lack of evidence from EEG and MRI, we could not identify the seizure-related pathological loci in the tested 70 partial epilepsy patients. So, by using DMN/BOLD-fMRI, we were able identify possible DMN changes in all tested partial epilepsy patients.

Currently, the application of ReHo analysis in partial epilepsy was limited. It is only used in children or primary epilepsy. For example, children with rolandic epilepsy (RE, also called BECTS) showed increased ReHo signals in the central, premotor and prefrontal region, and decreased ReHo signals in bilateral orbitofrontal cortex and temporal pore [25]. Untreated children absence epilepsy (CEA) showed increased ReHo in bilateral insula and decreased ReHo in DMN region [26]. In primarily generalized tonic-clonic seizures (GTCS), bilaterally and symmetrically altered ReHo was observed in the cortical and subcortical structures [27]. In this study, we focused on the ReHo alterations in adult partial epilepsy patients. Even we lack EEG and conventional MRI evidence for the pathologic regions in adult partial epilepsy patients; we could identify decreased ReHo in inferior parietal lobule, precentral and postcentral gyrus, thalamus, paracentral lobule, cerebellum anterior and posterior Lobe, and bilateral insula. The decreased ReHo signal in local neuron areas might be caused by the damages in neurology network and brain functions after seizure discharges. In this study, ReHo described the synchronization of local neuron activity in neuron network, while DNM reflected the concordance of separate functional connectivity brain region. When combining these two methods, we could evaluate the disease characters more comprehensively.

ReHo and DMN functional connectivity evaluate brain functions from two different aspects. While ReHo mainly describes the synchronization of regional neuron activity in neural network, DMN functional connectivity measures the synchronization among brain regions. The combination of these two technologies would provide a more complete evaluation of the neural network for epilepsy patients. In this study, epilepsy patients showed significant decreased ReHo in PCu, parietal lobe, frontal lobe, insula, thalamus and cerebellum, which is caused by the impairment in coordination of neuron activity within above brain region. ReHo findings suggest abnormalities in individual neural nodes. On the other hand, functional connectivity uses PCu as seed and the connectivity regions decreased significantly in parietal lobe, temporal lobe and Limbic Area, indicating obstruction of connecting pathway between PCu and above brain regions. There, ReHo combined with functional connectivity can evaluate both the intra-nodal activity and the inter-nodal connection of entire neural network.

As for the clinical symptoms, epilepsy patients showed significant lower concentration level in numerical depth test than health controls (Supplementary Table 1). Numerical depth test is used to evaluate patients’ concentration ability. Inferior parietal lobe is responsible for concentration and stimulus surveillance [28]. Epilepsy patients showed significantly decreased ReHo and functional connectivity in inferior parietal lobe, suggesting the dysfunction of inferior parietal lobe as the pathophysiology basis for attention drop in epilepsy patients. Moreover, decreased ReHo in PCu region suggests a dysfunction of PCu, which might be responsible for recurrent conscious disturbance [29–32]. Decreased ReHo in precentral gyrus suggests a decrease of coordination in precentral gyrus neurons, which is the pathology basis for deviated mouth and limb convulsion in epilepsy patients. In addition, decreased ReHo in insula is related to dysfunction of insula and deficiency of patients’ language fluency and logic memory [33–34]. Thalamus is another region with decreased ReHo and its dysfunction is related to deficient learning and memory activity [35–36].

Besides ReHo, region with decreased DMN functional connectivity is also related to clinical symptoms. Limbic Area is constituted of gyrus subcallosum, cingulated gyrus, parahippocampal gyrus, the deep surface of hippocampus dentate gyrus, and related amygdale and hypothalamus. Limbic Area is an important part to regulate inner organs, feelings and emotion, and to participate into advanced knowledge activities, such as study and memory. Previous research had indicated that epileptic discharge of abnormal DMN region in patients might be conducted to Limbic Area, and lead to functional connectivity disorders between hippocampal gyrus and DMN region [37]. In this study, the functional connectivity between parahippocampal gyrus and DMN decreased significantly, suggesting a comprehensive inhibition of neural network. This abnormality is associated with decreased cognitive function, depression and anxieties in partial epilepsy patients. Moreover, decreased DMN functional connectivity of left superior temporal gyrus is associated with memory disorders.

Taken together, based on our findings in ReHo analysis and DMN functional connectivity of brain regions, we conclude that both the abnormal ReHo regions and decreased DMN functional connectivity regions are related to clinical symptoms in partial epilepsy patients. Further study will focus on the potential correlation between ReHo signal and DMN functional connectivities.

In study, patients with partial epilepsy were not categorized into specific epilepsy types, such as temporal lobe epilepsy. In future study, we can focuse on more specific type of partial epilepsy patients and try to optimize parameters in DMN and ReHo methods. Besides DMN and ReHo, other statistic methods could be developed in future in epilepsy diagnosis.

## SUPPLEMENTARY MATERIALS FIGURES AND TABLES


