# Supplementary material for: Altered DMN functional connectivity and regional homogeneity in partial epilepsy patients: a seventy cases study
Source: Oncotarget. 2017 Aug 28;8(46):81475–84. doi: 10.18632/oncotarget.20575 (PMC5655301; doi:10.18632/oncotarget.20575)
Supplement: Supplementary file 1 [file oncotarget-08-81475-s001.pdf]

Altered DMN functional connectivity and regional homogeneity in partial epilepsy patients: a seventy cases report

SUPPLEMENTARY MATERIALS

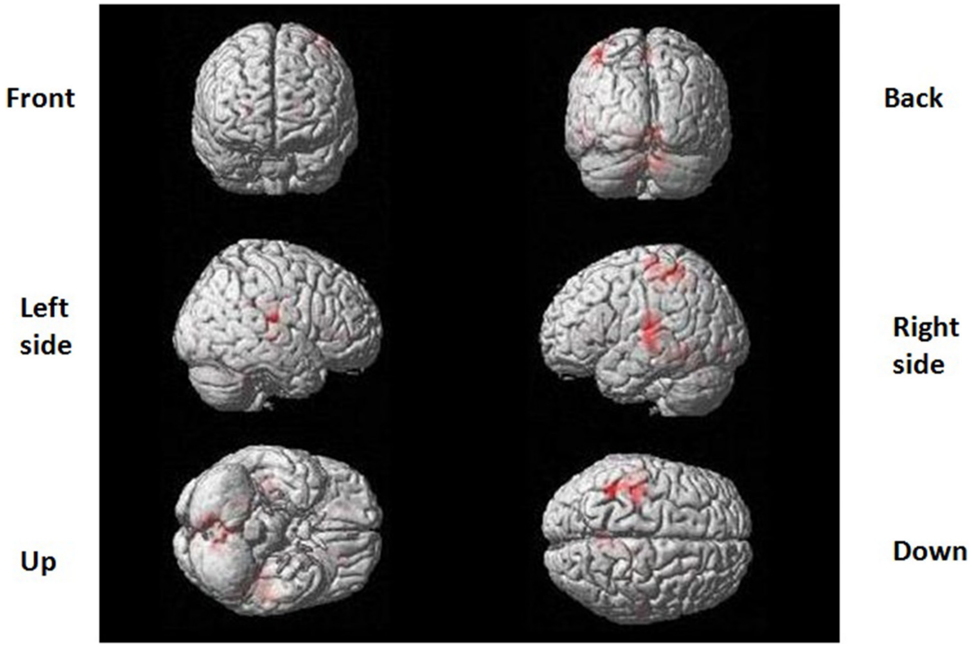

Supplementary Figure 1: 3-dimension projection images for decreased DMN functional connectivity regions in epilepsy patients and healthy controls. Red region indicate the decreased DMN functional connectivity regions.

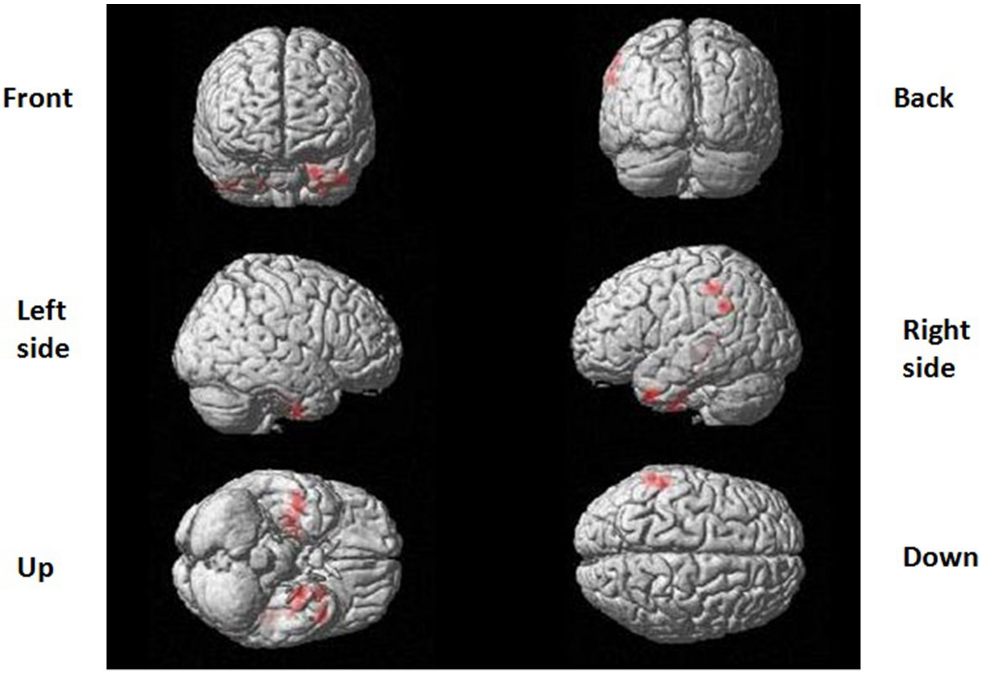

Supplementary Figure 2: 3-demension projection image for decreased ReHo regions in epilepsy patients. Red regions indicate the decreased ReHo.

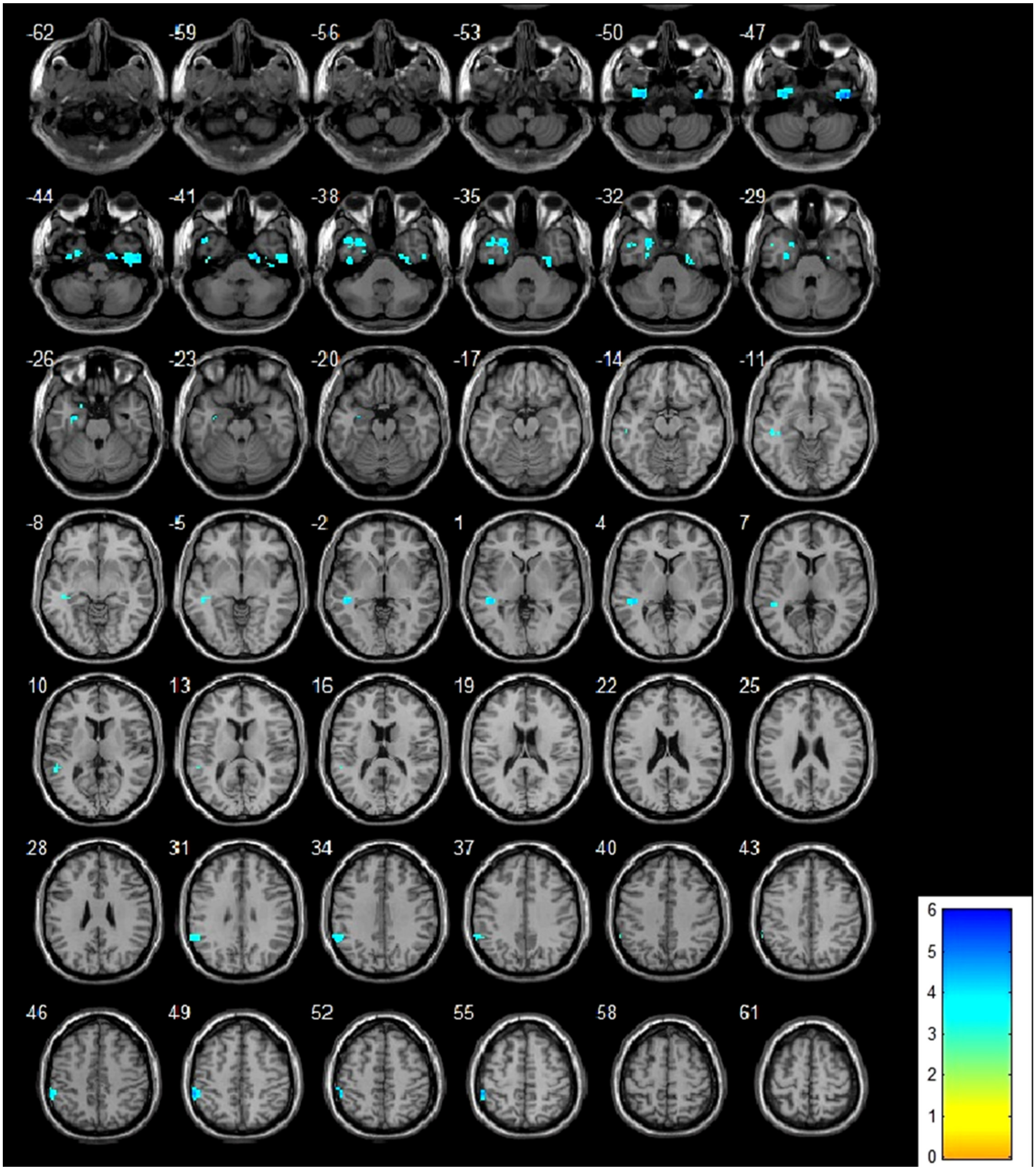

Supplementary Figure 3: Projection map on axial brain image 3-dimension projection images for decreased DMN functional connectivity regions in epilepsy patients, compared to healthy controls.

Supplementary Table 1: Comparison of the psychological assessment between patients and healthy controls

Variables		Participants		*p*-value
		Patients (n = 62)	Controls (n = 64)	
Numerical depth		11.92 ± 3.22	14.93 ± 1.94	< 0.001
Language fluency	Accuracy number	14.54 ± 3.87	22.11 ± 4.52	< 0.001
Logic memory	Immediate memory	18.38 ± 7.42	25.70 ± 8.59	< 0.001
	Prolonged memory	17.15 ± 6.58	23.89 ± 9.27	< 0.001
SDS score		47.77 ± 9.75	42.33 ± 9.43	0.007
SAS score		43.15 ± 10.12	37.59 ± 8.30	0.003

Supplementary Table 2: DMN Functional connectivity regions in epilepsy patients

Pixel size	L/R	Brain region	T statistics	Coordinate		
				x	y	z
1047	L	Precuneus	36.17	–12	–48	33
	L	Cingulate Gyrus	28.83	–6	–45	27
	L	Posterior Cingulate	27.66	–6	–45	15
61	L	Angular Gyrus	20.36	–45	–72	36

Supplementary Table 3: DMN functional connectivity regions in healthy control group

Pixel size	L/R	Brian regions	T statistics	Coordinate		
				x	y	z
3085	L	Precuneus	38.99	–12	–51	33
	L	Posterior Cingulate	36.19	–9	–45	21
	L	Posterior Cingulate	29.28	–3	–54	18
1409	L	Superior Frontal Gyrus	22.44	–9	57	36
	R	Medial Frontal Gyrus	16.01	6	48	–9
	L	Medial Frontal Gyrus	14.96	–6	63	18
518	L	Fusiform Gyrus	17.76	–48	–6	–27
	L	Middle Temporal Gyrus	16.68	–51	3	–36
	L	Inferior Temporal Gyrus	15.98	–54	–6	–33
779	R	Fusiform Gyrus	17.44	51	–3	–27
	R	Angular Gyrus	16.13	42	–60	30
	R	Superior Temporal Gyrus	15.41	57	–60	27
